# Comparison of information-theoretic to statistical methods for gene-gene interactions in the presence of genetic heterogeneity

**DOI:** 10.1186/1471-2164-11-487

**Published:** 2010-09-03

**Authors:** Lara Sucheston, Pritam Chanda, Aidong Zhang, David Tritchler, Murali Ramanathan

**Affiliations:** 1 Department of Biostatistics, State University of New York, Buffalo, NY 14260, USA; 2 Department of Cancer Prevention and Control, Roswell Park Cancer Institute, Buffalo New York, 14263, USA; 3 Department of Computer Science and Engineering, State University of New York, Buffalo, NY 14260, USA; 4 Department of Biostatistics, University of Toronto; 5 Ontario Cancer Institute, Toronto, Ontario, Canada, USA; 6 Department of Pharmaceutical Sciences, State University of New York, Buffalo, NY 14260, USA

## Abstract

**Background:**

Multifactorial diseases such as cancer and cardiovascular diseases are caused by the complex interplay between genes and environment. The detection of these interactions remains challenging due to computational limitations. Information theoretic approaches use computationally efficient directed search strategies and thus provide a feasible solution to this problem. However, the power of information theoretic methods for interaction analysis has not been systematically evaluated. In this work, we compare power and Type I error of an information-theoretic approach to existing interaction analysis methods.

**Methods:**

The *k-*way interaction information (KWII) metric for identifying variable combinations involved in gene-gene interactions (GGI) was assessed using several simulated data sets under models of genetic heterogeneity driven by susceptibility increasing loci with varying allele frequency, penetrance values and heritability. The power and proportion of false positives of the KWII was compared to multifactor dimensionality reduction (MDR), restricted partitioning method (RPM) and logistic regression.

**Results:**

The power of the KWII was considerably greater than MDR on all six simulation models examined. For a given disease prevalence at high values of heritability, the power of both RPM and KWII was greater than 95%. For models with low heritability and/or genetic heterogeneity, the power of the KWII was consistently greater than RPM; the improvements in power for the KWII over RPM ranged from 4.7% to 14.2% at for α = 0.001 in the three models at the lowest heritability values examined. KWII performed similar to logistic regression.

**Conclusions:**

Information theoretic models are flexible and have excellent power to detect GGI under a variety of conditions that characterize complex diseases.

## Background

Numerous complex diseases such as cancer, cardiovascular disease, mental illnesses, and autoimmune disorders are the result of interactions among many exogenous and endogenous factors operating on one or more biological pathways. However, reliably identifying the key underlying gene-gene (GGI) and gene-environment interactions (GEI) has proven difficult because the number of interactions increases combinatorially with the number of variables considered and resultant high dimensionality presents significant statistical challenges in interaction analyses.

Broadly, existing methods for analyzing GGI (and GEI) can be either parametric or non-parametric and can leverage dimensionality reduction or regression-based methodologies. Parametric approaches model explicitly the nature of the interaction, whereas the nonparametric approaches do not model these relationships. Multifactor Dimensionality Reduction (MDR) [1] and Restricted Partitioning Method (RPM) [2] are representative examples of dimensionality reduction methods whereas logistic regression [3] and logic regression [4] are examples of regression-based methods. Generalized MDR is a hybrid method that contains elements of both categories [5]. Logistic regression is used for GGI analysis by treating the genotype and genotype combinations as predictors in genetic models (e.g., dominant, additive) for categorical phenotypes.

Information theoretic methods are a promising and novel approach for identifying GGI and GEI, which do not require formulation and evaluation of specific interaction models. Information theoretic approaches such as AMBIENCE [6] employ directed search using entropy-based metrics and differ from dimensionality reduction methods such as MDR and RPM that utilize pooling into high and low-risk groups. Although some information theory-based methods have begun to emerge for interaction analysis, these methods have not been investigated sufficiently to gain widespread acceptance. For example, interaction dendrograms [7], an information theoretic visualization method and normalized mutual information [8] have been used with MDR [9] to investigate GGI and GEI. Previously we demonstrated the usefulness of the *k*-way interaction information (KWII), a multivariate information theoretic metric, for analyzing genetic association with both discrete and continuous phenotypes [6,10]. In this information theoretic framework, variable combinations with positive KWII values are operationally defined as interactions [6]. Information theoretic methods can be used for discrete phenotypes with more than two classes and their underlying formalism addresses the false associations that can be caused by the presence of linkage disequilibrium (LD) [6]. Information theoretic methods do not require an explicit model to be specified and can identify disease-associated GGI when multiple loci are involved. The mathematical properties of multivariate entropy measures can also be harnessed for the design of computationally efficient interaction analysis algorithms that do not require exhaustive search and can therefore enable the analysis of larger data sets [6].

Given the substantial differences between existing approaches and information theoretic methods and the potential applicability of the latter for genome-wide interaction analysis [6], there is a critical need for systematic and comparative assessment of the power and false positive rate of these methods. In this paper we assess power of our approach, MDR and RPM to detect GGI with and without genetic heterogeneity (GH); genetic heterogeneity adds a layer of complexity to interaction analysis and is a hallmark of many complex human diseases (e.g., Alzheimer's disease) and thus it is important to study the performance of methods under these conditions.

## Methods

### Description of the KWII Information Theoretic Method

#### Definition of Interaction

The *k*-way interaction information (KWII) is a parsimonious, multivariate measure of information gain, defined below [11,12]. We use the KWII as the measure of interaction information for each variable combination. We operationally define "A positive KWII value for a variable combination indicates the presence of an interaction, negative values of KWII indicates the presence of redundancy and a KWII value of zero denotes the net absence of K-way interactions".

Our information theoretic methods identify statistical interactions as determined by measurable changes in entropy.

#### Entropy

The entropy, *H(X)*, of a discrete random variable *X *can be computed from the probabilities *p(x) *using the formula:

$$H(X)=-\sum_{x}p(x)\log p(x)$$

#### *k-*way Interaction Information (KWII)

The KWII is presented as in [10]. For the 3-variable case, the KWII is defined in terms of the individual entropies of *H(A), H(B) *and *H(C)*, the lower order combinations, *H(AB), H(AC), H(BC) *and all three variables *H(ABC)*: *KWII*(*A*;*B*;*C*) = - *H*(*A*) - *H*(*B*) - *H*(*C*) + *H*(*AB*) + *H*(*AC*) + *H*(*BC*) - *H*(*ABC*). For the case of *K *genetic or environmental variables and phenotype variable *P *on the set *ν *= {*X*_1_, *X*_2_, ..., *X*_*K*_, *P*}, the KWII is written as an alternating sum over all possible subsets *T *of *ν *using the difference operator notation of Han [13]:

$$KWII(\nu )\equiv -\sum_{T\subseteq \nu }{\left(-1\right)}^{\left|\nu \right|-\left|T\right|}H(T)$$

The number of genetic and environmental variables *K *in a combination is called the order of the combination. The KWII represents the gain of information (positive values) or synergy between the variables, the loss of information (negative values) or redundancy between the variables or no change in information (values of zero) viewed as the absence of K-way interactions due to the inclusion of additional variables in the model. It quantifies interactions by representing the information that cannot be obtained without observing all *K *variables at the same time [11,12,14,15].

#### AMBIENCE Algorithm

AMBIENCE is an information theoretic search method and algorithm for detecting GEI that employs the KWII. The details of AMBIENCE are described in Chanda *et al. *[6].

### GGI Simulations

The power and proportion of false positives of the KWII in detecting GGI were compared to that of MDR, RPM, and Logistic Regression using three sets of simulations (Table 1). Two groups of simulations were performed in Set 1. First we compared power and type 1 error of KWII and MDR given models of disease heterogeneity with varying allele frequency, penetrance and heritability; GGI models were constructed using parameters as described in Culverhouse (Table 2) [2]. Second, we assessed power and type I error of KWII and RPM given varying allele frequencies, heritability and penetrance using GGI models with parameters identical to those of Richie *et al. *(Table 3) [2,16]. The second set of simulations compared the power and type I error of KWII with MDR, RPM, and logistic regression. We simulated a disease model with genetic heterogeneity (GH) combining the models of Culverhouse and Ritchie to evaluate the performance of these four approaches [2,16]. The third set of simulations was of a larger scale and was based on real genotype data. Simulated datasets consisted of 50,000 samples from the GAW15 problem 2 data set were expanded by incorporating GH models of Ritchie et al with varying allele frequencies, penetrances and heritabilities.

**Table 1:** Overview of simulation sets used to test power to detect GGI and type I error.

Model	Sample size	Number of SNPs	Number of Interactions	MAF	*K_p_*	*h^2^*
**Set 1A: Comparison of KWII to MDR^a^**						

1-GH			2	0.5	0.05	0.013
						
2-GH					0.025	
			
3-GH	400	10	2	0.25	0.06	0.007
						
3			1			0.03
			
4-GH			2	0.1	0.025	0.003
						
4			1			0.012

**Set 1B: Comparison of KWII to RPM^b^**						

5A					0.3	0.62
						
5B						0.3
						
5C						0.15
					
6A					0.1	0.22
						
6B	200	7	1	0.5		0.11
						
6C						0.056
					
7A					0.01	0.02
						
7B						0.01
						
7C						0.005

**Set 2: Comparison of KWII to MDR, RPM, and Logistic Regression Approaches^c^**						

1-GH and 7C	600, 1200 & 2400	10	3	0.5	0.037	0.013

**Set 3: GAW15 Problem 2^d^**						

1-GH	2400	865	2	0.5	0.05	0.013
						
2-GH					0.025	
		
3-GH		865	2	0.25	0.06	0.007
						
3			1			0.03
		
4-GH		865	2	0.1	0.025	0.003
						
4			1			0.012

GH: Genetic Heterogenity; *K_p _*= population prevalence; *h^2 ^*= Broad sense heritability;

^a ^Penetrance is modeled as in Table 2 [16]

^b ^Penetrance is modeled as in Table 3 [2]

^c ^*Kp *values for Interactions 1 and 2 are each 0.05 (penetrance table from Model 1-GH from [16]) and for interaction 3 *Kp *is 0.01 (penetrance table from Model 7C from [2])

^d ^[27]

**Table 2:** Penetrance tables for comparison of KWII to MDR.

*Model 1-GH* ***K***_***p ***_**= 0.05, *h***^**2 **^**= 0.013**				*Model 2-GH* ***K***_***p ***_**= 0.025, *h***^**2 **^**= 0.013**			
	*BB*	*Bb*	*bb*		*BB*	*Bb*	*bb*
*AA*	0.0	0.1	0.0	*AA*	0.0	0.0	0.1
*Aa*	0.1	0.0	0.1	*Aa*	0.0	0.05	0.0
*aa*	0.0	0.1	0.0	*aa*	0.1	0.1	0.0

***Models 3 and 3-GH*** ***K***_***p ***_**= 0.06, *h***^**2 **^**= 0.03 and 0.007**				***Models 4 and 4-GH*** ***K***_***p ***_**= 0.025, *h***^**2 **^**= 0.012 and 0.003**			

	*BB*	*Bb*	*bb*		*BB*	*Bb*	*bb*
*AA*	0.08	0.07	0.05	*AA*	0.07	0.05	0.02
*Aa*	0.1	0.0	0.1	*Aa*	0.05	0.09	0.01
*aa*	0.03	0.1	0.04	*aa*	0.02	0.01	0.03

The penetrance values are based on the models in [16].

**Table 3:** Penetrance tables comparison of KWII to RPM.

*Model 5: K_p _*= 0.3											
***Model 5A: h*^2 ^= 0.62**				***Model 5B: h*^2 ^= 0.30**				***Model 5C: h*^2 ^= 0.15**			

	*BB*	*Bb*	*bb*		*BB*	*Bb*	*bb*		*BB*	*Bb*	*bb*
*AA*	0.2	0.0	1.0	*AA*	0.23	0.09	0.79	*AA*	0.25	0.15	0.65
*Aa*	0.0	0.6	0.0	*Aa*	0.09	0.51	0.09	*Aa*	0.15	0.45	0.15
*aa*	1.0	0.0	0.2	*aa*	0.79	0.09	0.23	*aa*	0.65	0.15	0.25

***Model 6: K_p _*= 0.1**											

***Model 6A: h*^2 ^= 0.22**				***Model 6B: h*^2 ^= 0.11**				***Model 6C: h*^2 ^= 0.056**			

	*BB*	*Bb*	*bb*		*BB*	*Bb*	*bb*		*BB*	*Bb*	*Bb*
*AA*	0.0	0.0	0.4	*AA*	0.03	0.03	0.31	*AA*	0.05	0.05	0.25
*Aa*	0.0	0.2	0.0	*Aa*	0.03	0.17	0.03	*Aa*	0.05	0.15	0.05
*aa*	0.4	0.0	0.0	*aa*	0.31	0.03	0.03	*aa*	0.25	0.05	0.05

***Model 7: K_p _*= 0.01**											

***Model 7A: h*^2 ^= 0.020**				***Model 7B: h*^2 ^= 0.010**				***Model 7C: h*^2 ^= 0.005**			

	*BB*	*Bb*	*bb*		*BB*	*Bb*	*bb*		*BB*	*Bb*	*bb*
*AA*	0.0	0.0	0.04	*AA*	0.003	0.003	0.031	*AA*	0.005	0.005	0.025
*Aa*	0.1	0.02	0.0	*Aa*	0.003	0.017	0.003	*Aa*	0.005	0.015	0.005
*aa*	0.04	0.0	0.0	*aa*	0.031	0.003	0.003	*aa*	0.025	0.005	0.005

The penetrance values are based on the models in [2].

### Power and Proportion of False Positives in KWII, MDR, RPM and Regression Models

Power and proportion of false positives (PFP) of each of the methods were compared using 1000 independent repetitions of the simulation procedure.

#### Permutation-Based *p-*values of KWII

For each simulation step, the *p*-value of the KWII of each combination was determined using 100,000 permutations. The permutations for each combination were conducted independently of the other combinations. The permutation procedure provides the null distribution of the KWII, i.e., when the combination of variables was not association with the phenotype. The *p*-value for the combination was defined as the proportion of permutations with KWII values that were greater than or equal to the observed KWII.

#### PFP of KWII

The PFP was calculated as the ratio of the number of false combinations detected as significant to the total number of possible false combinations in 1000 replications of the simulation procedure. The total number of false combinations possible was computed to order 2 or less.

#### Power of KWII

KWII power was defined as the proportion of repetitions in which the combinations involved in GGI were identified as significant at the α-values of interest. A false combination was defined as a combination containing one or more SNPs that were not associated with the phenotype in the simulation model. Because there were no marginal effects in all of our simulated models, all one-SNP combinations are also false combinations.

For the KWII, power calculations were conducted for 28 closely spaced *p*-values from 0.01 to 0.001 in intervals of 0.001 and from 0.001 to 0.0001 in intervals of 0.0001 and from 0.0001 to 10^-5 ^in intervals of 10^-5^. Power of the KWII at α-values of 0.01, 0.001 and 0.0001 were obtained by interpolating the two PFP values that bracketed the α-value of interest.

#### MDR, RPM and Regression

Statistical significance for MDR models was obtained using the *R*^2 ^statistic generated by comparing the observed prediction error for each MDR model to the null distribution obtained from 10,000 permutations.

An interaction is deemed detected when the deviance of the full model [3] (see section on *Logistic Regression *below) from the model containing only the main effect terms is significant using the likelihood-ratio test with degrees of freedom equal to the difference in the residual degrees of freedom between the two fitted models.

The power and PFP for MDR, RPM, and logistic regression were obtained at nominal α-values of 0.01, 0.001 and 0.0001 corresponding to the KWII.

### Simulation Set 1A: Comparing KWII to MDR

The four two-locus models and simulation parameters (penetrance matrices, number of SNPs, allele frequency and sample size) employed in the original MDR power evaluation paper by Ritchie et al. [16] were used for comparison against the KWII. The design parameters and penetrance matrices for the models are summarized in Table 1 and Table 2, respectively. The MDR implementation was downloaded from http://sourceforge.net/projects/mdr/.

A case-control study design with 200 cases and 200 controls was assumed. Case control status was denoted with indicator variable, *C*. Ten diallelic SNPs were simulated. The allele frequency for all the SNPs in Models 1 and 2 was 0.5; for Models 3 and 4, the minor allele frequencies (MAF) for all SNPs were 0.25 and 0.10,respectively. Genotypes were assumed to be in Hardy-Weinberg equilibrium proportions.

Models 1-GH, 2-GH, 3-GH and 4-GH contained genetic heterogeneity (GH) with two pairs of interacting loci, *SNP*(*1*) with *SNP*(*2*), defined as Interaction 1 and *SNP*(*9*) with *SNP*(*10*), defined as Interaction 2. For all 4 GH models each Interaction increased risk in half of the cases. The corresponding penetrance matrices in Table 2 were used for simulations for both pairs of interacting loci. Models 3 and 4 contained only Interaction 1. The remaining SNPs were not associated with the phenotype. For each model, we simulated 1000 data sets.

### Simulation Set 1B: Comparing KWII to RPM

The penetrance matrices, number of SNPs, allele frequency and sample size for these comparisons were identical to those evaluated by Culverhouse [2]. Tables 1 and 3 summarize the design parameters (sample size, prevalence, *K_p _*and broad sense heritability, *h*^2^) and genotype penetrance matrices, respectively for the nine models [2]. The code for RPM was provided by Dr. Culverhouse.

A case-control study design with 100 cases and 100 controls was assumed. Case control status was denoted with indicator variable, *C*. Seven diallelic SNPs with equally frequent alleles were assumed for all SNPs in Models 5-7. Genotypes were assumed to be in Hardy-Weinberg equilibrium proportions. *SNP(1) *and *SNP(2) *were involved in the gene interactions that were associated with the disease phenotype variable; *SNP(3) *through *SNP(7) *were not associated. For each model, we simulated 1000 data sets

### Simulation Set 2: Comparing KWII to MDR, RPM and Logisitic Regression

The power and type I error of KWII was compared to that of MDR, RPM, and logistic regression under a more complex model of GH for varying study sizes.

#### Logistic Regression

Logistic regression models used to test for interaction are as outlined in Cordell [3]. The logistic model for a GGI interaction is written:

$$\begin{array}{c} \log\left(\frac{r}{1-r}\right)=\mu +a_{1}x_{1}+d_{1}z_{1}+a_{2}x_{2}+d_{2}z_{2}+\\ i_{aa}x_{1}x_{2}+i_{ad}x_{1}z_{2}+i_{da}z_{1}x_{2}+i_{dd}z_{1}z_{2} \end{array}$$

where, *r *is the probability of each individual being a case, *μ *corresponds to the mean effect, the terms *a_1_*, *d_1_*, *a_2_*, *d_2 _*are the dominance and additive effect coefficients of the two SNPs, *i_aa_*, *i_ad_*, *i_da_*, *i_dd _*represent their product coefficients and *x_i _*and *z_i _*are dummy variables with *x_i _*= 1, *z_i _*= -0.5 for one homozygous genotype (*AA *or *BB*), *x_i _*= 0, *z_i _*= 0.5 for the heterozygous genotypes (*Aa *or *Bb*), and *x_i _*= -1, *z_i _*= -0.5 for the homozygous genotypes (*aa *or *bb*). This model was expanded to capture the multiple SNP interactions that characterized these simulations.

We assumed a case-control study design with an equal number of cases and controls for three sample sizes, 600, 1200, 2400. Case control status was denoted with indicator variable, *C*. Ten equal frequent diallelic SNPs in Hardy Weinberg Equilibrium proportions were modeled.

Case status was determined by three pairs of interacting loci (*Interaction 1*-*SNP*(*1*) with *SNP*(*2*); *Interaction 2 *-*SNP*(*5*) with *SNP*(*6*) *and Interaction 3 -SNP*(*9*) with *SNP*(*10*)), with each pair increasing risk in one-third of the cases. The penetrance matrix of Model 1-GH was used for *Interaction 1 *and *Interaction 2 *and the penetrance matrix of Model 7C was used for *Interaction 3*. The remaining four SNPs, *SNP*(*3*), *SNP*(*4*), *SNP*(*7*), *SNP*(*8*), were not associated with the phenotype. The penetrance matrices obtained from the simulations are shown in Table 4 for the combinations of SNP pairs involved in *Interactions 1, 2 *and *3*. Power was assessed from 1000 independent repetitions of the simulation procedure as previously described.

**Table 4:** Penetrance tables for comparing KWII to the other four competing methods.

*Interaction 1* *SNP(1) with SNP(2)*				*Interaction 2* *SNP(5) with SNP(6)*				*Interaction 3* *SNP(9) with SNP(10)*			
	*BB*	*Bb*	*bb*		*BB*	*Bb*	*bb*		*BB*	*Bb*	*bb*
*AA*	0.02	0.053	0.02	*AA*	0.02	0.053	0.02	*AA*	0.035	0.035	0.042
*Aa*	0.053	0.02	0.053	*Aa*	0.053	0.02	0.053	*Aa*	0.035	0.038	0.035
*aa*	0.02	0.053	0.02	*aa*	0.02	0.053	0.02	*aa*	0.042	0.035	0.035

The overall disease prevalence is *K_p _*= 0.037. Only pairwise penetrances for SNP directly involved in *Interactions *1, 2, and 3 are shown. The penetrance values for Interaction 1 and Interaction 2 are from [16] whereas those for Interaction 3 are from [2].

### Simulation Set 3: Application of KWII Method to a Larger Dataset with Real Genotypes

Given the unavailability of publicly accessible real datasets with validated GGI in order to assess the performance of the KWII approach in the presence of real genotypes, we employed a hybrid approach in which simulated interactions were planted in the context of the real genotypes in the GAW15 problem 2 data set. The data were obtained from http://www.gaworkshop.org/ and used with permission. We selected SNPs spanning a 10 Kb region of chromosome 18 q containing a dense panel of genotypes for 2300 SNPs in 920 samples. The data were pre-processed to remove samples with missing data and SNPs that were not in Hardy-Weinberg equilibrium (χ^2 ^test at α = 0.05). The method of Carlson et al. [17] was then used to select a set of SNPs with an LD threshold of *R^2 ^*= 0.9. We refer to this data set as the GAW15-P2 data set.

We generated a population of 50,000 individual genotypes by resampling with replacement from the GAW15-P2 data.

The six models assessed were those from Simulation set 1a. For Model 1-GH and Model 2-GH, we identified the SNPs with MAF of 0.5 ± 0.01; for Model 3 and 3-GH, we identified SNPs with MAF of 0.75 ± 0.01 and for Model 4 and 4-GH, we identified the SNPs with MAF of 0.90 ± 0.01.

For a pair of SNPs, SNP *i *and SNP *j*, for each individual in the population, the case-control status was randomly assigned based on the penetrance matrix for the interaction models of interest with the genotypes of SNP *i *and SNP *j*. Relative risk was set to 2.0 and 1200 cases and 1200 controls were then selected for analysis. This process was repeated for 100 random pairs of SNPs selected for each model.

Power was defined as the proportion of repetitions for which the interacting SNP pairs had the highest values of KWII. For the models with GH, two second-order combinations with the highest KWII values were considered; for models without genetic heterogeneity, only the second-order combination highest KWII value was considered.

## Results

### Visualizing KWII Values in GGI Models Without Main Effects

Ritchie et al. [16] and Culverhouse [2] conducted detailed power and type I assessments of MDR and RPM models, respectively, to detect gene interactions without main effects. In these models, the phenotype variation is not attributable to any of the individual loci but is explained by the combined presence of two or more loci (i.e., there are no marginal effects). We investigated the characteristics of the KWII metric in each of the two-locus gene interactions models from the Ritchie et al. [16] and Culverhouse [2] reports.

**Figure 1:**
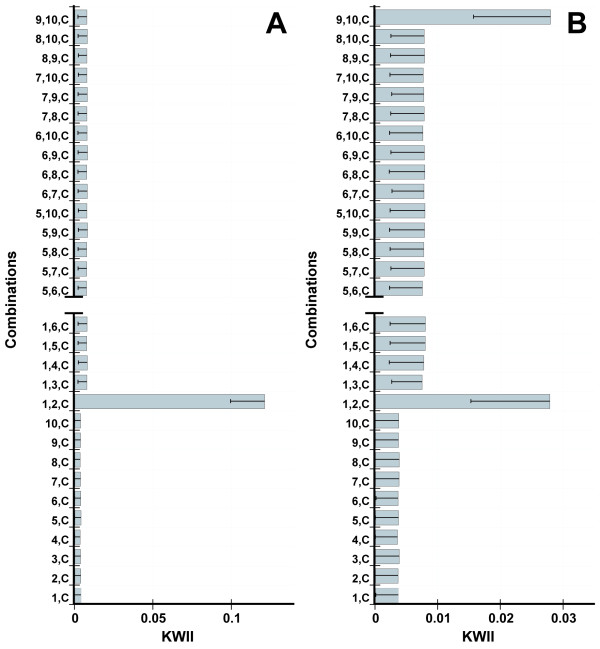
**Figure 1A and B show the KWII spectra corresponding for Model 3 and Model 3-GH**. Note the *x*-axis scales differ between Figures 1A and B. To improve clarity, a subset of uninformative combinations is not included in the plot; this is indicated with the break. The error bars are standard deviations.

Figures 1A and 1B summarize the KWII for different combinations of SNPs for Model 3 and Model 3-GH, which were among the models used for comparing KWII to MDR in Simulation set 1A. These two models have a MAF of 0.25 and vary in heritability as well as the number of underlying susceptibility loci contributing to case status. Both plots contain prominent peaks for the informative two-SNP combination {*1*, *2*, *C*}, which contains both SNPs, *SNP(1) *and *SNP(2) *involved in Interaction 1. Peaks corresponding to combinations containing only SNPs that are not associated with the phenotype or the single SNP combinations {*1*, *C*} or {*2*, *C*} are not present. In the presence of GH in Model 3-GH, an additional peak corresponding to combination {*9*, *10*, *C*} is present and the peak height of combination {*1*, *2*, *C*} is reduced. The heritability *h^2 ^*decreases from 0.03 (Model 3) to 0.007 (Model 3-GH) in presence of GH with the prevalence *K_p _*remaining constant at 0.06. Figure 1A and 1B effectively demonstrate the characteristics of the simulated data, i.e., absence of main effects, the impact of a decrease in heritability on the metric and the presence of genetic heterogeneity.

Thus, the KWII can be used to visualize information regarding the GGI combinations including the presence of GH and is also, as expected, sensitive to a reduction in information content of a combination that would occur with changes in penetrance and allele frequency.

### Simulation Set 1A: Power and Type I Error Comparison of KWII to MDR

In Table 5, we show the power of the KWII to MDR to detect both Interaction 1 {*1*, *2*, *C*} and Interaction 2, {*9*, *10*, *C*} for α-values of 0.01, 0.001 and 0.0001. The power of the MDR and KWII methods to detect Interaction 2 alone was similar to their power to detect Interaction 1 alone and is therefore not shown.

**Table 5:** Power and proportion of false positive comparison of the KWII to MDR.

Model	α	Interaction 1		Interactions 1 & 2*		MDR PFP
			
		KWII	MDR	KWII	MDR	
	0.01	98.7	19.9	98.1	0.7	0.0047
**1-GH**	0.001	94.3	1.3	89.4	0.9	0.0003
	0.0001	85.6	0.6	72.9	0.4	0.0002

**2-GH**	0.01	100	36.0	100	61.7	0.0116
	0.001	99.7	12.2	99.5	33.9	0.0029
	0.0001	98.1	5.0	96.3	23.3	0.0013

**3**	0.01	100	91.3	-	-	0.0254
	0.001	100	56.1	-	-	0.0112
	0.0001	100	33.1	-	-	0.0067

**3-GH**	0.01	58.3	5.3	32.3	1.5	0.0028
	0.001	28.2	1.4	8.2	0.6	0.0010
	0.0001	15.3	0.1	2.2	0.3	0.0001

**4**	0.01	99.6	54.0	-	-	0.0164
	0.001	97.5	8.0	-	-	0.0042
	0.0001	91.5	0.5	-	-	0.0010

**4-GH**	0.01	48.2	0.7	22.1	2.0	0.0019
	0.001	19.6	0	3.4	0.6	0.0005
	0.0001	9.1	0	0.9	0.3	0.0001

*Models 3 and 4 had only a two SNP interaction (Interaction 1) present and thus power values are not applicable.

The simulations are based on models in [16].

For all models in this simulation set, the power of KWII was greater than that for MDR and KWII was more robust to the presence of GH than MDR. The greatest difference in power between the two approaches was seen for Model 1-GH and Model 2-GH. For both of these models the power of KWII was greater than 90% for α values as low as 0.001. The power of both approaches was substantially reduced when GH was introduced into Models 3 and 4. Given two 2 SNP interactions contributing equally to disease for α = 0.001, the power of MDR decreased to almost zero while KWII faired better with power at ~30% and 20% for Model 3-GH and Model 4-GH, respectively.

### Simulation Set 1B: Power and Type I Error Comparison of KWII to RPM

Table 6 summarizes the power and type I error for GGI models for different values of population prevalence (*K_p_*) and the heritability (*h^2^*).

**Table 6:** Comparison of the power and proportion of false positives of KWII to RPM.

*K_p_*	*h^2^*	Model	α	Power %		RPM PFP*
					
				KWII	RPM	
0.3	0.62	5A	0.001	100	100	0.0010
			0.0001	100	100	0.0002
	
	0.3	5B	0.001	100	100	0.0018
			0.0001	100	100	0.0004
	
	0.15	5C	0.001	97.7	93.0	0.0014
			0.0001	90.1	84.2	0.0003

0.1	0.22	6A	0.001	100	100	0.0011
			0.0001	100	100	0.0002
	
	0.11	6B	0.001	100	100	0.0014
			0.0001	100	100	0.0004
	
	0.056	6C	0.001	86.6	75.2	0.0013
			0.0001	73.6	56.5	0.0002

0.01	0.02	7A	0.001	100	100	0.0010
			0.0001	100	100	0.0002
	
	0.01	7B	0.001	99.3	98.4	0.0013
			0.0001	98.4	95.8	0.0003
	
	0.005	7C	0.001	72.6	58.4	0.0016
			0.0001	51.6	40.5	0.0005

* **α **is calibrated to the empirical false positive rate of KWII

The simulations are based on the models in [2].

For all A and B models the KWII and RPM had excellent power, greater than 98% for both α-values, to detect GGI. For the lowest *h^2 ^*values, Models 6C and 7C, the power of the KWII was 17.1% (11.4%) and 11.1% (14.2%) greater than that of RPM at α = 0.0001 (α = 0.001), respectively.

### Power and Proportion of False Positives for Simulation Set 1

**Figure 2:**
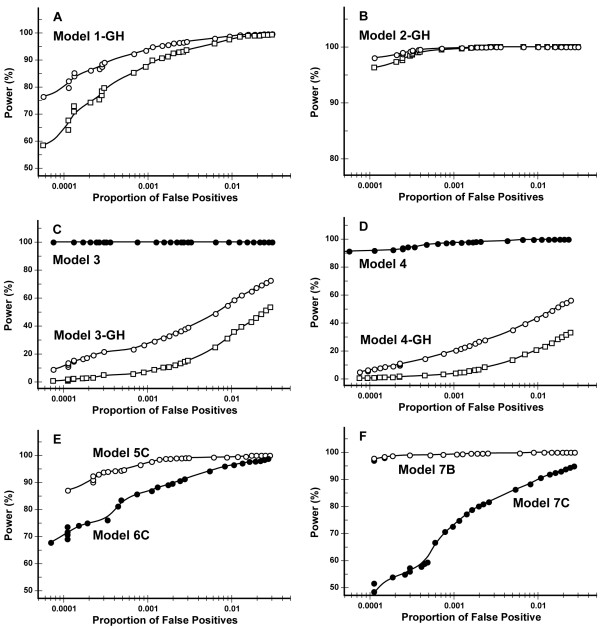
**Figure 2A-F are receiver-operating characteristic plots showing the dependence of the power of the KWII on proportion of false positives for models 1-GH, 2-GH, 3, 3-GH, 4, 4-GH, 5C, 6C and 7A-7B in Table 1**. Models 5A, 5B, 6A, 6B and 7C had power greater than 99% over the range of proportion of false positives examined and are not shown. The open circles in Figure 2A-3 D represent the power for detecting one of the two interacting pairs of loci and the open squares represent the power for detecting both loci. The filled circles in Figures 2C and 2 D correspond to the corresponding model without genetic heterogeneity whereas in Figures 2E and 2F the filled circles are used to distinguish between the different models. The power of the KWII at α-values of 0.001 and 0.0001 are summarized in Table 5 and 6.

Figure 2 graphically summarizes the relationships between the power and the proportion of false positives using receiver-operator characteristic (ROC) curves of the KWII for each of the models examined in Simulation set 1A and 1B. The power of the KWII to detect the individual interacting pairs in Model 1-GH (Figure 2A) was 90% with the proportion of false positives of 0.0004. Both interacting pairs of loci in Model 2-GH (Figure 2B) and the interacting loci in Model 3 (Figure 2C) were identified with power of greater than 95% at the lowest proportions of false positives values obtained. As expected GH, decreasing heritability and allele frequency reduces the power of KWII to detect disease susceptibility loci in the simulated data.

### Simulation Set 2: Power and Proportion of False Positives Comparing KWII to MDR, RPM and Regression Approaches

The studies of the power of MDR [16] and RPM [2] used small sizes of 200 and 100 subjects per group, respectively which are atypical for interaction studies. To address this, we compared the KWII to four competing methods, MDR, RPM, logistic regression and logic regression for total sample sizes of 600, 1200 and 2400 containing an equal number of cases and controls for α = 0.001 and 0.0001. Data was simulated such that case status was attributable to three pairs of interacting loci with penetrance matrices from the MDR [16] and the RPM papers [2].

**Figure 3:**
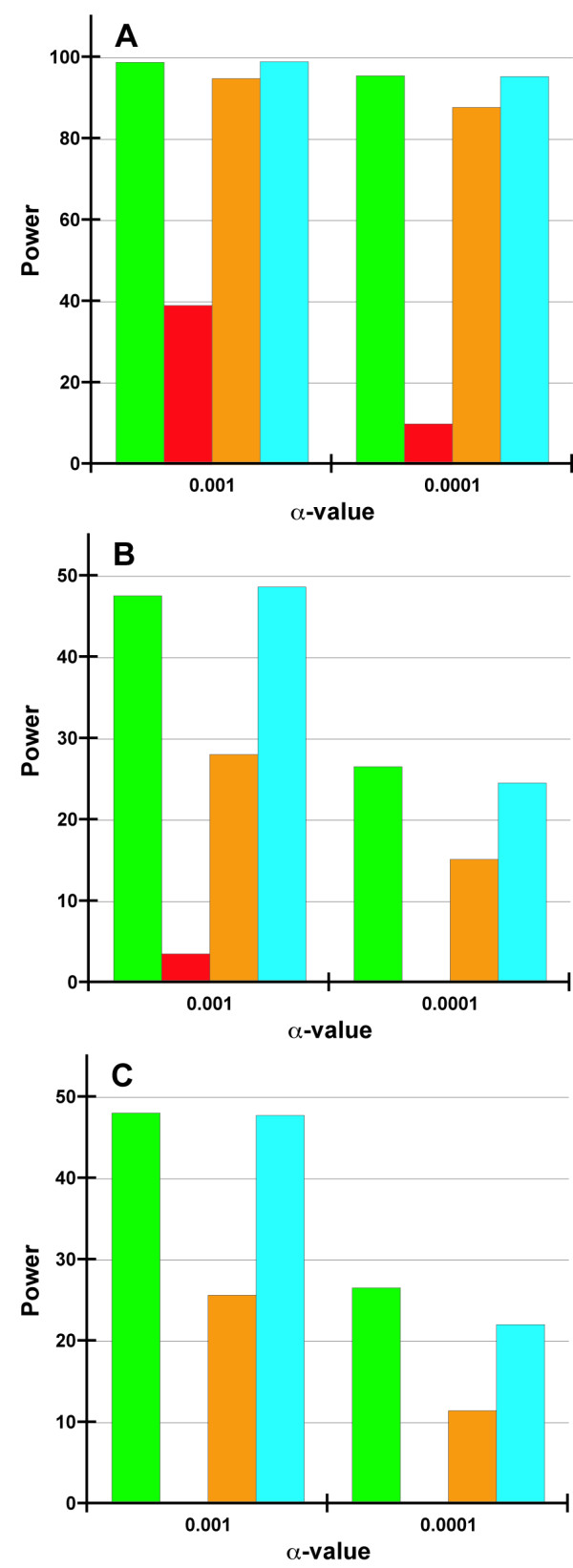
**Figures 3A-C compare the power of KWII (green bars) to that of MDR (red bars), RPM (orange bars) and blue for logistic regression at α-values of 0**.001 and 0.0001. The sample size was 1200. Figure 3A corresponds to *Interaction 1*, Figure 3B corresponds to Interaction 3 and Figure 3C represents the power to detect all three interactions. The penetrance matrices for the combinations of SNP pairs involved in *Interactions 1, 2 *and *3 *are shown in Table 4. The bar corresponding to MDR in Figure 3C is apparently not visible because the power was low.

Figure 3A-C compares the power of KWII to MDR, RPM and logistic regression at a sample size of 1200 for α = 0.001 and 0.0001. Table 7 compares the power and proportion of false positives of the KWII method to MDR, RPM, and logistic regression across the sample sizes of 600, 1200 and 2400. The power was calculated for each of the three interacting pairs of SNPs and as an overall power for all three interactions.

**Table 7:** Comparison of the power and false positive proportions of KWII to MDR, RPM, and regression approaches.

Interaction	Sample Size	α	Power %			
			
			KWII	MDR	RPM	Logistic
**Interaction 1**	600	0.001	68.9	7.8	48.8	68.3
		0.0001	42.2	3.7	29.5	43.3
	
	1200	0.001	98.9	39.1	94.9	99.1
		0.0001	95.6	10.0	87.8	95.4
	
	2400	0.001	100	65.3	100	100
		0.0001	100	13.8	100	100

**Interaction 3**	600	0.001	15.1	0.04	7.2	14.8
		0.0001	4.8	0.03	3.2	5.2
	
	1200	0.001	47.6	3.6	28.1	48.7
		0.0001	26.6	0.04	15.2	24.6
	
	2400	0.001	95.1	6.6	84.4	94.0
		0.0001	83.5	0.05	70.8	84.2

**All 3 Interactions**	600	0.001	7.6	0.01	2.2	7.5
		0.0001	15.2	0.01	0.5	14.1
	
	1200	0.001	48.1	0.03	25.7	47.8
		0.0001	26.6	0.01	11.5	22.1
	
	2400	0.001	95.1	2.9	84.4	94.0
		0.0001	83.5	0.01	70.8	84.2

**Interaction**	**Sample Size**	α*	**Proportion of False Positives**			
			
			**KWII**	**MDR**	**RPM**	**Logistic**

**All 3 Interactions**	600	0.001	-	0.0021	0.0010	0.0013
		0.0001	-	0.0010	0.0001	0.0001
	
	1200	0.001	-	0.0075	0.0013	0.0016
		0.0001	-	0.0011	0.0003	0.0002
	
	2400	0.001	-	0.0103	0.0009	0.0013
		0.0001	-	0.0014	0.0002	0.0002

*α is calibrated to the empirical false positive rate of KWII.

The model used for the simulations contained three interacting pairs of SNP as summarized in Table 1: *Interaction *1 and *Interaction *2 in were based on [16] whereas *Interaction *3 was based on [2].

For Interactions 1 and 3, the differences between the methods were most apparent at the lowest value of sample size, *n *= 600. For both Interaction 1 and Interaction 3, the KWII method and logistic regression had the highest power, followed in order by RPM, logic regression and MDR. For all methods, the power values for Interaction 1 were generally higher greater than those for Interaction 3. Not surprisingly, the power to detect all three interactions generally followed the power of the method to detect Interaction 3. The results for Interaction 2 were similar to those for Interaction 1 as the two pairs interactions were based on the same penetrance table and therefore the results are not shown.

These results highlight the power of the KWII method and demonstrate that it has comparable or greater power than a diverse range of competing methods.

### Simulation Set 3: Application of KWII Method to a Larger Dataset with Real Genotypes

We used the GAW15-P2 data set to assess the power of the KWII in the context of a larger-scale data set containing real genotypes. Our methodology incorporated known interactions planted in the context of real genotypes to overcome the lack of real data sets with experimentally validated examples of the gene-gene interactions. Quality control filtering and tag SNP selection yielded 895 individuals genotyped at 865 SNPs of which 23, 22 and 23 had minor allele frequencies of 0.1 ± 0.01, 0.5 ± 0.01 and 0.25 ± 0.01 respectively. We assessed power of the KWII at a sample size of 2400 (1200 cases and 1200 controls) for Model 1-GH, Model 2-GH, Model 3, Model 3-GH, Model 4 and Model 4-GH.

For all GH Models power was consistently highest for detecting Interaction 1 and lowest for detecting both interactions; power to detect Interaction 2 was within 1% - 3% of that to detect Interaction 1 for all GH modes. For the GH models power to detect Interaction 1 (both interactions) ranged from 74% (48%) in Model 4-GH to 91% (84%) in Model 2-GH. The power to detect GGI in models without GH, Models 3 and 4, was 100% and 99%, respectively.

## Discussion

We examined the power and proportion of false positives of the KWII against a diverse group of multi-locus methods that included MDR, RPM, logistic regression and logic regression, demonstrating that the power of KWII metric is greater than MDR, RPM and logic regression and comparable to logistic regression for a class of realistic models both with and without genetic heterogeneity. To our knowledge, this is the first detailed comparison of power and false positive proportion comparisons between existing interaction analysis approaches and those based on information theory.

The power of KWII exceeded the power of MDR for all models in Simulation sets 1 and 2. The discrepancy in power is attributable to differences in the algorithms. KWII has greater power than MDR because it selects *all *significant combinations separately while MDR selects only the best model, such that if two or more combinations of the same order are associated with a phenotype, as in the case of GH, MDR selects only one of them. In addition to the inability to detect the independent genetic contributions to models of GH MDR can be dependent on higher order combinations for power. This is illustrated by Model 2-GH; the power of MDR to detect both Interaction 1 *and *Interaction 2 is greater than its power to detect the interactions individually. This dependence coupled with the fact that MDR uses an exhaustive search approach also means that MDR would be very computationally inefficient for larger datasets as the number of combinations increases combinatorially with number of variables and combination order [18]. MDR is being continuously improved and used to analyze quantitative phenotypes and family data [19-21], computational efficiency remains the rate-limiting factor irrespective the improvements [22,23]. Cattaert et al. [19] have developed FAM-MDR method, which addresses correlation between observations in family-based studies and extends the model based MB-MDR approach [24] to handle continuous covariates and continuous phenotype. In contrast with the classical MDR, FAM-MDR considers multiple multi-SNP models for significance evaluation. Further research on extending the KWII based approach to handle family data is ongoing.

For Simulation sets 1 and 2, we found that RPM has reduced power when compared to KWII. When working with quantitative outcomes, RPM uses variances of the trait for each genotype group for merging groups of genotypes with closest mean trait values. While this works well for quantitative traits this approach does not translate as well for case-control data for particularly for less frequent diseases. This effect is compounded when only a small proportion of the variance of the trait is explained by genetics (Simulation 1B, Model 7C). While RPM performed reasonably well in models without GH, when GH was introduced (Table 7, Interaction 3) the ability of RPM to properly partition based on the proportion of cases for a given genotype is hampered because multiple different loci are contributing equally to disease. This is evidenced by a reduction in power, which is only overcome by substantially increasing the sample size.

In Simulation set 2, it is clear that logistic regression and KWII have almost identical power (within ~1% for all sample sizes and alpha values). Logistic regression models were also run for Simulation set 1B (results not shown) and again KWII and logistic models were powered within 2% of one another for all simulations; one approach was not consistently better than another. Despite similar power, the two methods differ in their model fitting approach. In logistic regression, model parameters are fit simultaneously but with the KWII approach, higher-order interactions are inferred after investigating and eliminating lower-order contributions. While we did not run models to detect power for three way interactions, Marchini *et al. *[25] found that loci with specific 3-way interactions are more likely to be detected by looking for two-locus effects. Thus while power is equivalent when considering two-locus models with and without GH, the genetic contribution to complex disease is proving to be oligogenic. Because KWII looks at increasing orders of interaction from low to high the method is well suited to finding these higher order combinations because it finds the lower order ones first.

For Simulation set 3, we employed a hybrid approach in which simulated interactions were planted in the context of the real genotypes from the GAW15 Problem 2 data set. This approach has the advantage of overcoming the limitations imposed by the lack of real data sets with well-validated interactions. Although there is a strong interest in detecting genes distantly located, and using a dense panel of SNPs spanning a 10 Kb region of single chromosome is not optimal, it could be argued that this approach is appropriate for post-genome wide studies (i.e., sequencing, deep genotyping). Furthermore SNPs selected from different chromosomal regions would have less LD amongst themselves, which may make interaction detection feasible with more traditional statistical approaches.

While KWII performed equivalently to the statistical gold standard (logistic regression) for the simple two locus models, with potential for greater power to detect higher order interactions, the method does have some limitations. KWII has sensitivities to missingness, sample size, low MAF and LD. As the number of missing genotypes increases in a dataset the estimation of entropies becomes more inaccurate. However given genotype imputation is regularly used for both candidate gene and genome wide studies this is easily remedied. As with statistical approaches such as MDR, RPM and regression low sample size reduces power when estimating higher order interactions, particularly when SNPs with very low allele frequencies are involved. Additionally KWII permutations have slightly higher false positive rate than logistic and MDR, although not substantially different. Lastly, the power of KWII to detect GGI is reduced when LD between the causal variant and the genotyped (or imputed) variant is low. However current chip and Hapmap coverage is quite good and this is problematic for all statistical methods used to test for allelic association

The results of these simulations provide clues as to how to perhaps more effectively search for interacting loci. One possible approach when working with a large dataset would be to combine our approach with logistic regression by running the AMBIENCE algorithm first with an anti-conservative alpha and then testing the combinations obtained using logistic regression models. This would be an improvement upon the computational speed of logistic regression and yield lower false positive rates than using KWII alone. This two stage approach is similar in spirit to that suggested by both Hoh *et al. *[26] and Marchini *et al. *[25] in which a liberal alpha is set for testing interactions in stage one in order to find SNPs with small marginal effects but large interaction effects.

Higher-order interactions are computationally intensive because of the rapid growth of the number of combinations. The power to detect higher order interactions is also limited because the number of samples within each multi-locus genotype stratum is a limiting factor. Given the challenges associated with higher-order interactions, it may be preferable to base model building on multiple lower-order interactions. Alternatively given pathway information from Gene Ontology (GO) or KEGG for example, AMBIENCE [6] could spend more computation time testing SNPs within the same pathway than across pathways. This will help to identify biological meaningful epistatic interactions that could then be analyzed using logistic models.

**Conclusions: **In this article, we compare an information theoretic approach with existing statistical methods to test for GGI and find that our method has excellent power and to detect interaction in both simulated and real data.

## Authors' contributions

All authors read and approved the final manuscript. LS and PC contributed to experimental design, data analysis and manuscript preparation. AZ contributed research resources and to project design. DT contributed to experimental design and manuscript preparation. MR contributed to project and experimental design, data analysis and manuscript preparation.
